# Mechanical and Thermophysical Properties of Carbon Fiber-Reinforced Polyethersulfone

**DOI:** 10.3390/polym14142956

**Published:** 2022-07-21

**Authors:** Valerii G. Torokhov, Dilyus I. Chukov, Victor V. Tcherdyntsev, Galal Sherif, Mikhail Y. Zadorozhnyy, Andrey A. Stepashkin, Ilya I. Larin, Elena V. Medvedeva

**Affiliations:** 1 Laboratory of Functional Polymer Materials, National University of Science and Technology “MISIS”, Leninskii Prosp, 4, 119049 Moscow, Russia; vgtorohov@gmail.com (V.G.T.); dil_chukov@mail.ru (D.I.C.); eng_galal_emad@mu.edu.eg (G.S.); priboy38@mail.ru (M.Y.Z.); a.stepashkin@misis.ru (A.A.S.); 2 Center for Genetics and Life Science, Department of Biomaterials, Sirius University of Science and Technology, 354349 Sochi, Russia; larin.ii@talantiuspeh.ru; 3 Institute of Electrophysics, Ural Branch, Russian Academy of Science, Amudsena Str., 106, 620016 Yekaterinburg, Russia; lena_p@bk.ru

**Keywords:** composite materials, carbon fibers, polyethersulfone, surface modification, interfacial interaction, adhesion, structure, thermal conductivity, thermal expansion

## Abstract

In this study, the mechanical and thermophysical properties of carbon fiber-reinforced polyethersulfone are investigated. To enhance the interfacial interaction between carbon fibers and the polymer matrix, the surface modification of carbon fibers by thermal oxidation is conducted. By means of AFM and X-ray spectroscopy, it is determined that surface modification changes the morphology and chemical composition of carbon fibers. It is shown that surface modification dramatically increases the mechanical properties of the composites. Thus, flexural strength and the E-modulus of the composites reinforced with modified fibers reached approximately 962 MPa and 60 GPa, respectively, compared with approximately 600 MPa and 50 GPa for the composites reinforced with the initial ones. The heat deflection temperatures of the composites reinforced with the initial and modified fibers were measured. It is shown that composites reinforced with modified fibers lose their stability at temperatures of about 211 °C, which correlates with the glass transition temperature of the PES matrix. The thermal conductivity of the composites with different fiber content is investigated in two directions: in-plane and transverse to layers of carbon fibers. The obtained composites had a relatively high realization of the thermal conductive properties of carbon fibers, up to 55–60%.

## 1. Introduction

Due to the combination of low density and excellent mechanical properties, carbon fibers (CFs) are one of the most promising reinforcers for polymer composites, especially in the fields of lightweight application [1,2,3]. Such composites have been extensively utilized as structural materials in the aeronautic and automotive industries [4]. Additionally, CF-reinforced polymer composites can be applied as antifriction materials [5,6], as thermal conductive materials for heat exchangers and the thermal management of compact electronic units [7,8], as electromagnetic shielding materials [9,10], as sensors for structural health monitoring [11,12], as ballistic protection materials [2,13], as high-strength self-healing materials [14,15,16], as materials for additive manufacturing [17,18], etc.

CF-reinforced polymer composite characteristics depend strongly on the shape of the fibers and their location in the polymer matrix. Short CF-reinforced composites are suitable materials for tribological and electromagnetic shielding applications [3,5,6,8,10,19,20]. The formation technology of such composites is cheap and easy enough [21,22]; short CFs can be distributed in a polymer matrix uniformly or in an oriented manner. Short CF-filled polymers exhibit good mechanical properties [23]; however, the excellent mechanical properties of CFs in polymer matrices cannot be realized completely in such composites. Thus, to achieve the mechanical characteristics required for high-performance structural application, continuous fibers are used for reinforcement.

Among the various types of continuous CFs, 2D CF fabrics are most commonly used as reinforcers for polymer composites designed for structural applications. Various types of CF fabrics are used in polymer composites; however, most applications of this kind are based on woven fabrics [3]. Twill weave is one of the most commonly used weave CF structures, providing optimal mechanical characteristics for polymer-based composites [24,25,26,27]. Moreover, twill weave CFs are widely utilized as the model object in theoretical works [28,29], and can be a suitable object to design new polymer-based composites.

Currently, most CF-reinforced composites used in a wide range of industries, including aerospace, wind turbines, sports equipment, and the automotive industry, are based on thermoset polymers such as epoxy resin. The wide application of such composites creates a problem of their recycling; the irreversible cross-linked structure of cured thermoset polymers results in significant difficulties in the recovery of the CFs and the thermosetting polymer, since they cannot be melted, remoulded, reprocessed, or re-crosslinked into solid polymers. The effective recycling of CF-reinforced thermosetting polymers is a now serious problem, requiring an elaboration of complex processing technologies, including thermal, mechanical, and chemical treatments [30,31,32]. Therefore, the elaboration of GF-reinforced composite materials with matrices based on easily recyclable thermoplastic polymers is being actively investigated. Thermoplastic-based composites show considerably higher static fracture toughness compared with thermoset (epoxy) composites [3]. Additional advantages of thermoplastics are their unlimited shelf life, low curing duration, the ability to correct defects and damage by reheating, the possibility of reforming defective products [33], high environmental resistance, and high chemical resistance, including to aviation fuels and oils [1].

In recent years, thermoplastic-based CF-reinforced composites have been elaborated and investigated widely [34]. High-performance polymers are of particular interest among thermoplastics due to their high thermal stability and mechanical properties. Polyethersulfone (PES) is a superior performance engineering plastic with a high glass transition temperature Tg of 225 °C and an operating temperature up to 180 °C. PES possesses several advantages, including high toughness, the possibility to produce and form complex shapes, good antifriction properties, high modulus and strength, good fatigue resistance and dimensional stability, good fire retardancy, and chemical and radiation resistance.

As well as other high-performance polymers, PES is widely used as a toughening modifier for the epoxy matrix in CF-reinforced composites. The addition of PES allows for the significant improvement of interlaminar fracture toughness in epoxy-based CF-reinforced composites in comparison with composites containing no PES [35,36,37,38]. Moreover, the addition of temperature-resistant PES to epoxy matrices can increase the fire resistance of CF-reinforced composites [39]. In addition to epoxy-based composites, it has been reported that an addition of PES to the poly(phthalazinone ether sulfone ketone) thermoplastic matrix reduces the melting viscosity of the matrix polymer and improves the properties of the CF-reinforced composite, including interlaminar fracture toughness and flexural strength [40].

A significant number of papers on CF-reinforced PES-based composites have been published in recent decades; however, most of them relate to composites containing short or unidirectional CFs. In [41,42], the mechanical and tribological properties of PES reinforced with short CFs and short glass fibers (GFs) are investigated. It was observed that the addition of both CF and GF increased the mechanical properties of PES, but the effect was higher in case of CF-reinforced PES. In terms of the tribological characteristics, a positive effect was observed only for CF-reinforced composites, and the addition of GF resulted in an increase in the friction coefficient magnitude [41]. In [42], a positive synergetic effect of the simultaneous reinforcement of PES with both short CFs and short GFs on the tensile/flexural strength and the wear resistance of the composites was observed. In [43], the mechanical properties of PES reinforced with aligned (oriented) short CFs were studied; it was shown that fracture of such composites occurred only after the formation of a dense network of internal cracks. PES reinforced with chaotically oriented short CFs showed a good concentration stability of tensile strength and a high effectiveness of electromagnetic interference shielding [44]. In [45], the rheological characteristics of PES reinforced with short CFs were studied to develop the optimal parameters of such composites’ additive manufacturing.

As it is known, since CF surfaces are chemically inert, the surface modification of CF is required to improve fiber adhesion with polymers. One of the modification methods consists of surface coating CF with epoxy resin [46,47]; it has been found that coating short CFs with phenol formaldehyde resin can simultaneously improve the tensile and flexural characteristics of PES-based composites [47]. Another method of modification is surface coating short CFs with graphite oxide, which can provide enhanced mechanical properties [48,49] and significantly improve the dimensional stability of PES-based composites in wide temperature range [50].

Unidirectional CF-reinforced composites are suitable objects to investigate the interface interactions between polymer matrices and reinforcers [51,52,53]. The comparison of the mechanical properties of several thermoplastic matrices, including PES reinforced with unidirectional CF, has shown that the nature of the polymer matrix significantly affects the interfacial shear strength value [52]. In [53], two types of PES with differential molecular weight were used as matrices in CF-reinforced composites. It was shown that PES molecular weight has nearly no effect on the flexural modulus and shear strength value, whereas the increase in molecular weight of the matrix polymer results in a significant rise in flexural strength and interlamellar fracture toughness. As the interface interactions are strongly affected by chemical bonding, the effects of unidirectional CF surface modification on PES-based composite properties have been studied. In [54], the influence of CF surface modification by thermal oxidation and by epoxy resin coating on the properties of unidirectional CF-reinforced PES is studied. It was found that the interfacial shear strength value of the composites was higher in the case of the epoxy resin modification of CF. The modification of the CF surface by carboxylic [55] and polyamic [56] acids has also been reported as an effective way to improve interfacial adhesion in PES-based composites reinforced with unidirectional CF.

Unfortunately, there are only a few papers on CF fabric-reinforced PES-based composites. In [57], it is reported that unidirectional CF fabric-reinforced PES showed excellent antifriction properties in comparison with epoxy resin-based composites. In [58,59,60], PES reinforced with twill weave CF fabrics treated with nitrogen and nitrogen oxygen cold plasma was investigated; it was found that the plasma treatment of CF resulted in an improvement in the tribological and mechanical properties of composites.

It is known that, due to the less permeable structure of CF fabrics, the polymer melt impregnation technique is not effective for the formation of composites with strong adhesion between the fibers and the matrix. For instance, in [58], the melt impregnation of twill weave CF with PES with various molecular weights was studied. It was observed that the mechanical properties of the obtained composited improved with a decrease in PES molecular weight, i.e., with an increase in the PES melt flow index. This means that composite properties in this case are limited by poor fiber/polymer adhesion, restricting the mechanical properties of polymers with higher molecular weight in composites formed by melt impregnation. To achieve better adhesion on the fiber/polymer interface, the polymer solution impregnation method can be used. Cyclopentanone [53] and dichloromethane [59,60] have been used as solvents for PES to form PES/CF composites. Recently [61,62,63], we used N-methyl-2-pyrrolidone as a solvent to obtain polysulfone-based composites reinforced with CF fabrics. In the present study, an elaborated technique is used to produce PES-based composites.

Thus, the purpose of this study is to develop a method of obtaining PES-based composite materials reinforced with carbon fabrics using solution technology, and to investigate the properties of the obtained materials. Moreover, since the interfacial interaction between the phases of the composites must be sufficient to provide good mechanical and thermophysical properties, the influence of the surface modification of carbon fibers on the properties of the obtained composites is studied.

## 2. Materials and Methods

Twill weave carbon fabric marked by 3K-1200-200 with a specific weight of 200 g/m^2^ and 3000 filaments in one thread (HC Composite, Moscow, Russia) was used as the reinforcement material. Ultrason E2020 P SR Micro polyethersulfone with a density of 1.37 g/cm^3^, a melt flow index of about 7.0 to 8.7 g/10 min, and a viscosity number of 59 in the form of powder (BASF, Ludwigshafen, Germany) was used as the matrix. For the proper impregnation of carbon fibers without breaking the reinforcement structure, PES powder was dissolved in N-2-methylpyrrolode (Eastchem, Changzhou, China) in a concentration of 20 wt. %. This method of impregnation has been considered as sufficient by some researchers [40]. Dissolution was conducted at room temperature, using a magnetic stirrer. The concentration was chosen from the best correlation between viscosity and the lower content of solvent. Moreover, impregnation with the solution of the aforementioned concentration allowed the impregnation of the fibers relatively quickly, with an even distribution of matrix.

**Figure 1:**
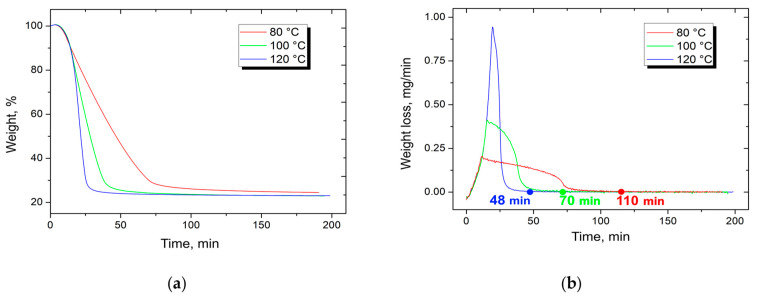
TGA curves, showing (**a**) residual weight percentage and (**b**) weight loss during evaporation of solvent from 20 wt. % PES/N-methyl-2-pyrrolidone solutions.

To remove the solvent from the matrix of the composite material, drying for 4 h at a temperature of 100 °C was conducted. The time and temperature of drying process were chosen as a result of thermogravimetric analysis (TGA). TGA was performed with TA Instruments Q600 (TA Instruments Inc., New Castle, DE, USA). During the experiment, samples were heated in alumina crucible to temperatures of 80, 100, and 120 °C, with a heat rate of 10 °C/min in a flow of air. The results of experiment are shown in Figure 1. The TGA analysis shows that at the temperature of 100 °C, after 70 min of the experiment, almost all solvent was evaporated from the sample. Therefore, we dried the prepreg sheets for 4 h or more in order to guarantee the absence of solvent in the prepreg sheets.

**Figure 2:**
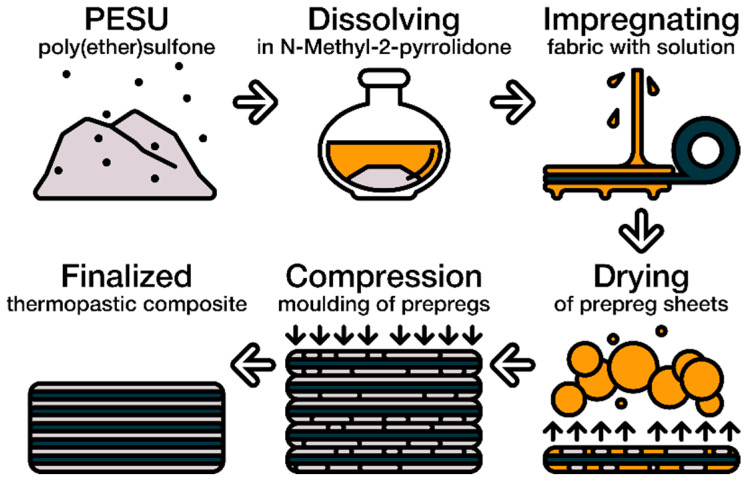
Scheme of obtaining of composite materials.

Obtained by the molding of sliced prepregs at 350 °C for 30 min under a pressure of 10 MPa; samples of the composite materials with fiber contents of 70, 60, 50, and 40 wt. % (marked as 70/30, 60/40, 50/50, and 40/60, respectively) were cut to a testable shape. The mold with the samples was cold in the atmosphere of air. These molding conditions are commended as best for PES by Ultrason. Figure 2 shows the scheme for obtaining of the composite material.

**Figure 3:**
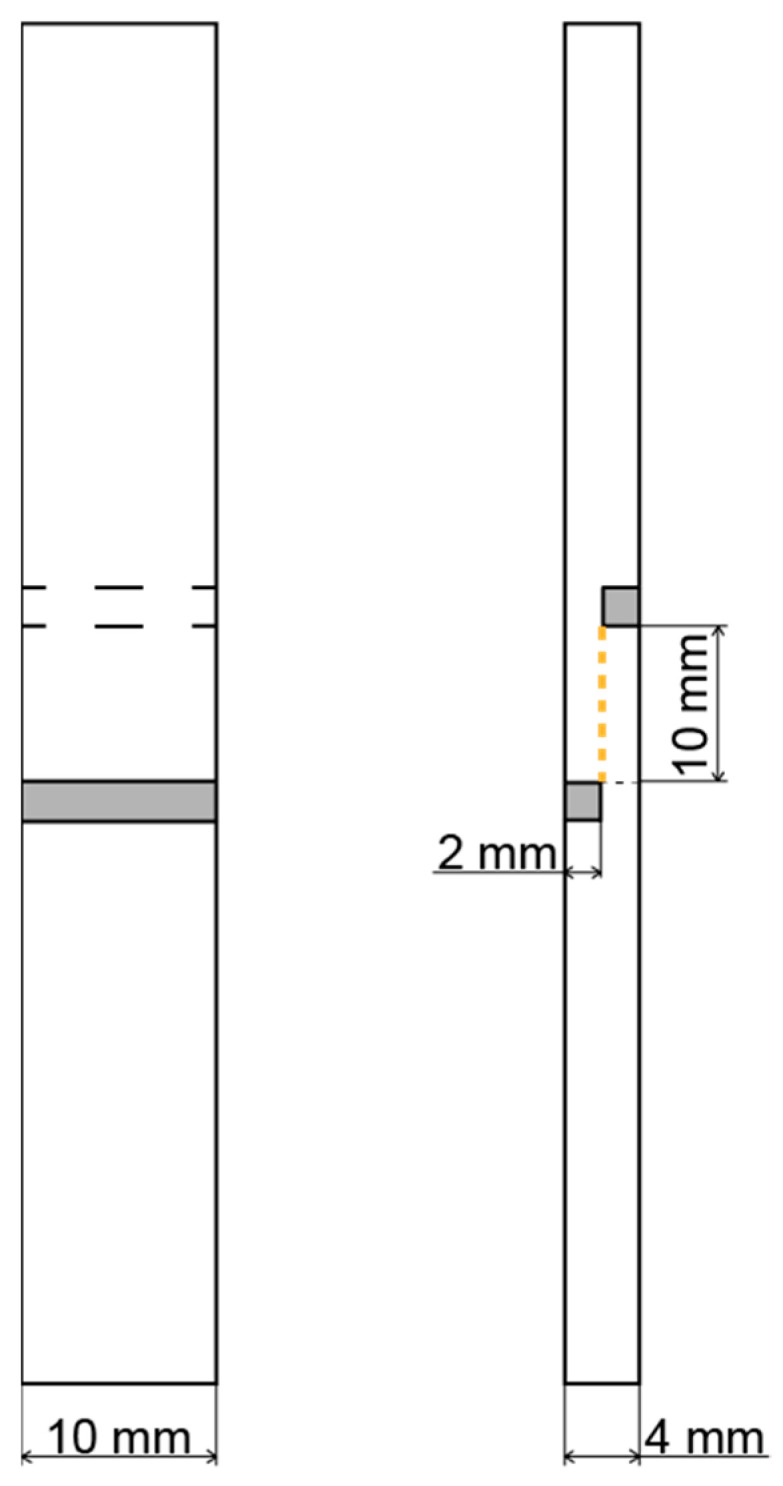
Geometry of samples for interlaminar shear strength tests.

Samples with thicknesses of 2 mm and 1 mm were used for the three-point bend test and the tensile strength test, respectively. These tests were performed according to the DIN EN ISO 14125-2011 and ISO 527:2021 standards, respectively. The width of the samples was about 10 mm. In both cases, a traverse speed of 10 mm/min was maintained. The adhesion between the matrix and the reinforcement material was learned by the ASTM D 3846-08 (2015) interlaminar shear test. To provide the interlaminar shear, specimens of special geometry were prepared. Figure 3 illustrates the scheme for the geometry of the used samples. Notches on both sides of the specimen were made with a Jet JMD-X1 milling machine, which allowed only one layer to be loaded during the test. Therefore, during the compression of the testing machine (traverse speed 1.3 mm/min), interlaminar shear occurred (orange line). All mechanical experiments were conducted with the Zwick/Roell Z020 (Zwick GmbH & Co., Ulm, Germany) universal testing machine at room temperature.

The surface modification of carbon fibers by thermal oxidation was chosen as the method for increasing the interphase interaction between the fibers and the polymer. Carbon fibers were heated and kept in an ambient environment at temperatures of 300, 400, and 500 °C for 30 min in a SNOL muffle furnace (SNOL Ltd., Velikie Luki, Russia). During this article, such fibers will be marked as TO300, TO400 and TO500, respectively. Heated and initials fibers were studied with the AIST-NT Smart SPM atomic force microscope (AIST-NT Inc., Novato, CA, USA). X-ray photoelectron spectroscopy with PHI5500VersaProbeII (Physical Electronics Inc., Chanhassen, MN, USA) was conducted for investigating the types of chemical bonds occurring on the surface of the fibers after modification.

The inner structure of the composites was studied by means of scanning electron microscopy (SEM) with VEGA 3 TESCAN (SEM photos of cracks samples after flexural stress tests) and TESCAN VEGA COMPACT microscopes (SEM photos of etched samples). To obtain SEM photos of the crack samples after the flexural stress tests, the samples were metalized by the magnetron sputtering of a 10 nm thick platinum layer at an amperage of 30 mA.

**Figure 4:**
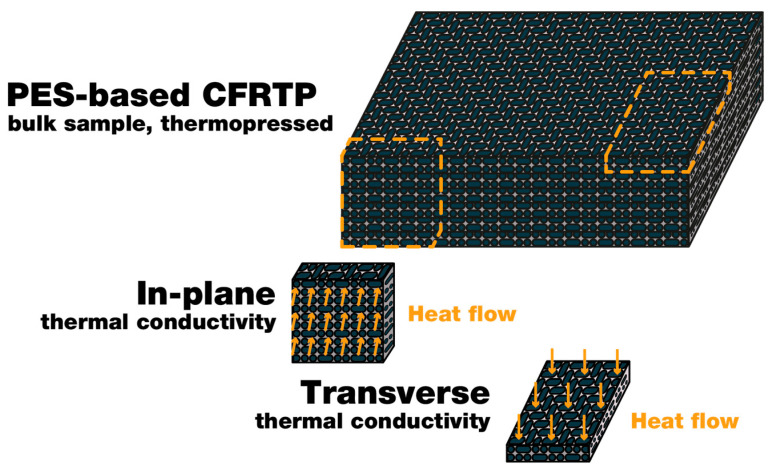
Principle scheme for measuring thermal conductivity of composites.

The thermophysical properties of the obtained composites were identified by dynamic mechanical analysis (DMA). The experiment was conducted using TA Instruments Q800 (TA Instruments Inc., New Castle, DE, USA). The experiment was conducted in the range of temperatures from 25 °C to 220 °C with a heating rate of 2 °C/min; a deformation of 0.1% with a frequency of 1 Hz was applied. The heat deflection temperatures of composites with different reinforcement levels were determined with CEAST HDT 3 VICAT (CEAST S.p.A., Pianezza, Italy). HDT experiments were performed in a silicone bath with initial load of 1.8 MPa and a heating rate of 120 °C/h. The thermal conductivity of the composites was studied in the temperature range from 298 to 498 K in accordance with ASTM E1461-13(2022) (Standard Test Method for Thermal Diffusivity by the Flash Method via Netzsch LFA 447 Netzsch GmbH, Selb, Germany). The study was carried out using square-shape specimens with a thickness of 1.5 mm and a length of each side of 8 mm. Samples were studied in two directions: parallel to the planes of composites and transverse to them. The principle scheme of the experiment is illustrated in Figure 4.

The density of the obtained samples was measured in water by the hydrostatic weighing method using an analytical balance of GR-202 (A&D Company Ltd., Tokyo, Japan) and the density determination was set at AD-1653. TA Instrument Q800 was used to estimate the thermal expansion coefficient.

## 3. Results and Discussion

**Figure 5:**
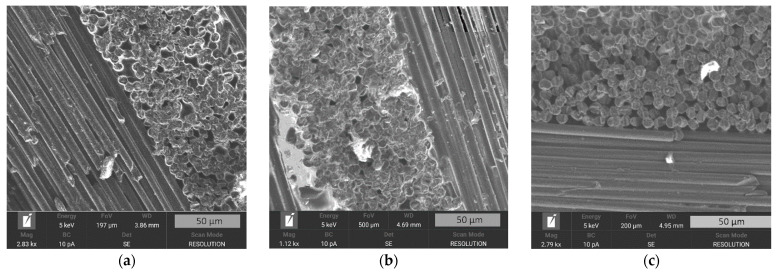
SEM photos of the etched surfaces of composite materials containing (**a**) 50 % wt. (**b**) 60 % wt. and (**c**) 70 % wt. of unmodified carbon fibers.

Figure 5 illustrates the structure of the obtained composite material with different fiber content. It is shown that the fiber-to-polymer weight ratio has a strong influence on the internal structure of the composite material. Thus, carbon fibers in a composite with a fiber content of 50 wt. % are fully arranged with the matrix polymer. A similar finding can be seen in Figure 5b, where the 60/40 composite is presented. In both Figure 5a,b polymer interlayers are visible, which is not peculiar for a composite with a fiber content of 70 wt. % (Figure 5c). Thus, it can be stated that fiber content strongly influences the structure of composite material.

**Figure 6:**
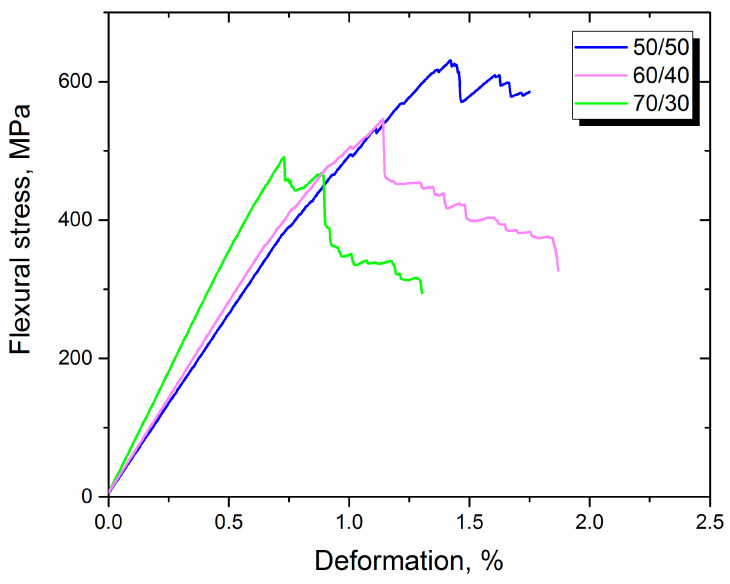
Typical stress–strain curves of composites reinforced with initial CF with different fiber content.

To identify the best composition of composite material from a mechanical point of view, mechanical tests were conducted. It was found that the composite with a fiber content of 50 wt. % performed the best, reaching 602 ± 23 MPa of flexural strength. The results of the three-point bend test can be seen in Figure 6.

Analyzing the typical stress–strain curve highlights a regularity of special interest. Firstly, the diagram is linear until it reaches a stress of about 400–450 MPa, which means that the material is elastic-deformed and no residual stresses have occurred, despite the thermoplastic nature of the matrix of the composite. Secondly, the E-modulus of the sample rises with rising fiber content. Such a tendency is explained by the higher E-modulus of fibers compared with the PES matrix. The higher content of fibers leads to a more rigid structure of the composite. Despite the highest E-modulus, the sample with the fiber content of 70 wt. % had the lowest flexural strength, which was about 482 ± 28 MPa. The insufficient quantity of the matrix in such a composite explains the observed behavior. Since the matrix in composite materials distributes appearing loads through reinforced fibers, a lack of polymers causes the incomplete relaxation of appearing loads, which leads to the early destruction of the composite material. From this perspective, a composite with a fiber content of 50 wt. % is optimal. When it comes to the fiber content at 40 wt. %, carbon fiber is lacking and the distribution of loads become insufficient again. The results of the flexural strength tests of the composites reinforced with the initial fibers are shown in Table 1.

In several research studies, interlaminar shear stress has been used as an effective method of the assessment of interfacial interaction in polymer composite materials [64,65,66]. The test showed the same regularities that were found after the bending tests. The results of interlaminar shear test are shown in Table 2.

Samples with 50 wt. % fiber content were optimal, reaching a shear strength of 20 MPa. A downward trend in shear strength with an increase in fiber content was observed, proving the aforementioned explanation of the existing tendencies. Accordingly, it was shown that a lesser quantity of polymer matrix could not provide sufficient adhesion in the composite materials.

Mechanical tests showed that the optimal fiber content was 50 wt. %, so it was decided to continue the experiments with only this amount of fiber content. To investigate the influence of the surface modification of carbon fibers by thermal oxidation on the adhesion and mechanical properties, a new iteration of mechanical tests for composites reinforced with modified carbon fibers was conducted.

**Figure 7:**
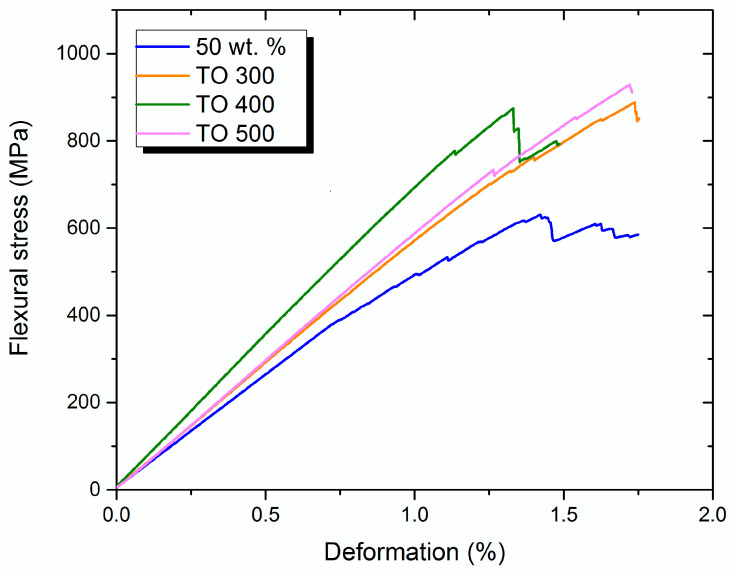
Typical stress–strain curves of 50/50 composites reinforced with modified carbon fibers during three-point bending test.

Figure 7 shows the typical stress–strain curves of the composites reinforced with modified fibers, compared with the composites reinforced with the initial CF (fiber content 50 wt. %).

Apart from flexural strength, which reached about 902, 932, and 962 MPa for the composites reinforced with TO300, TO400, TO500 fibers, respectively, the E-modulus raised significantly and was approximately 60 GPa, compared with the initial 50 GPa. Moreover, differences in the mechanisms of destruction and crack spread could be noted. The stress–strain curve for the composite reinforced with initial fibers contained a lot of sharp drops, indicating a crack break in the layers of the composite. Such behaviors of destruction occur when adhesion between the matrix and the reinforcement is insufficient, or loads are not distributed evenly through the fibers. The stress–strain curves for composites reinforced with modified fibers were straight, showing that the cracks opened instantaneously. Tension increases are distributed until a critical amount of energy opens a crack and destroys a sample.

**Figure 8:**
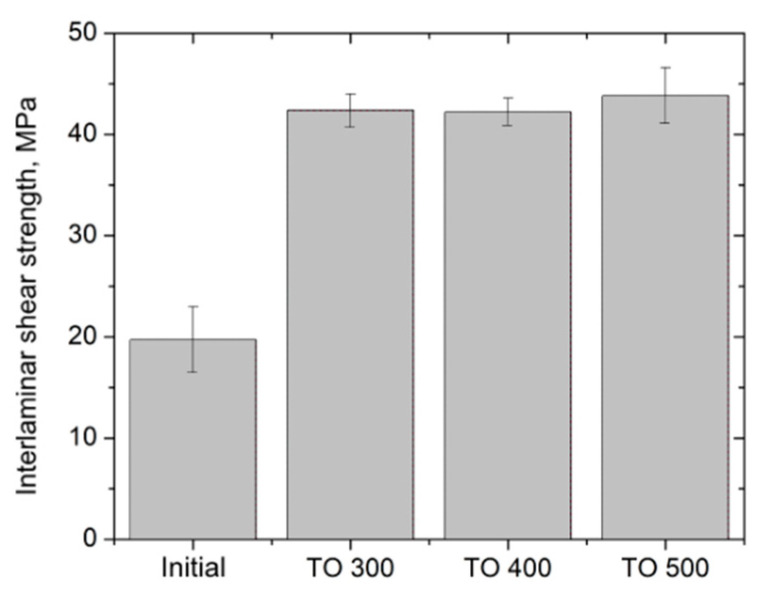
Results of interlaminar shear strength test of 50/50 composites reinforced with modified carbon fibers.

Interlaminar shear tests showed that the surface modification of carbon fibers resulted in a better interfacial interaction between the matrix and the reinforcement. The results of the experiment are shown in Figure 8.

Interlaminar shear strength raised remarkably, reaching about 40 MPa for composites reinforced with modified fibers, which is almost two times higher than the shear strength of the composites with the initial CF.

For the clarification of the mechanism of improvement for the adhesion between the phases of the composite material, X-ray photoelectron spectroscopy and atomic force microscopy of the carbon fibers was conducted.

**Figure 9:**
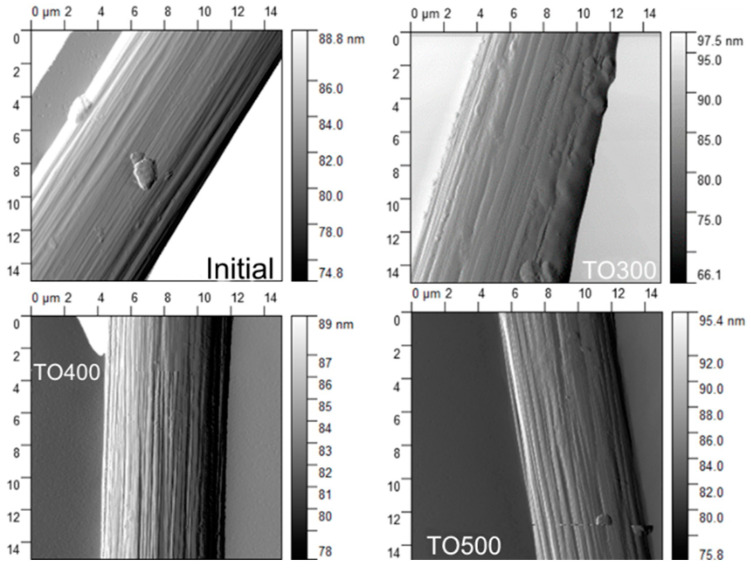
AFM photos of carbon fibers before and after surface modification.

The analysis of AFM allowed us to investigate the differences in the morphology of carbon fibers after surface modification. The AFM results are presented in Figure 9. The photo of the initial filament represents the coated surface of the fibers with shallow furrows between the fibrils. After thermal oxidation by heating to 300 °C, coagulation and a change in the structure and morphology of the polymer coating of the carbon fiber occurred, which resulted in the appearance of uneven hills and lowlands on the surface of the filament. The surface modification at 400 °C and 500 °C changed the morphology of the surface of fibers dramatically and seemed to completely remove the coating from the surface of the fibers, exposing the clear surface of the filaments with deep furrows, compared with the initial fibers. Moreover, after oxidation at 500 °C, the furrows became deeper, which can be explained by the oxidation and evaporation of amorphous carbon, which is located between the fibrils.

As it has previously been reported [61,62,63], the surface modification of carbon fibers by thermal oxidation can significantly change the chemical composition of the surfaces of CFs. Here, we show that atomic concentrations of carbon, oxygen, nitrogen, and some other less-comprised elements on the surfaces of CFs changed dramatically depending on the temperature of thermal oxidation. As almost all coatings are made of polymers which contain oxygen (in order to have better adhesion with epoxy resin), the atomic concentration of oxygen can be considered as an indicator of the presence of a sizing agent. After thermal oxidation at 300 °C, the amount of oxygen remained the same; consequently, the coating was not removed after this treatment. Oxidation at the temperature of 500 °C removed the coating completely as the concentration of oxygen dropped. Fluctuations in nitrogen content can be explained by the oxidation of coating after 300 °C treatment, and the further oxidation of amorphous carbon at 500 °C with the release of residual nitrogen.

Surface modification also changes the composition of chemical bonds on the surface of the fibers and controls the adhesion between the reinforcement and the matrix polymer. Compared with the functional groups on the surfaces of the initial fibers, which were CHx, C-O and -COO-, the quantity of the forementioned groups reduced after thermal oxidation at 300 °C. Reduction was interpreted as the oxidation and evaporation of the coating of the CFs. After oxidation at 500 °C, new functional groups occurred such as C=O, -COOH, C-OH, and others, which could be a result of the total decomposition of the coating on the surface.

Consequently, it can be concluded that surface modification improves the interfacial interaction between carbon fibers and PES by the development of surface morphology and the increase in the quantity of functional groups on the surfaces of the fibers by the decomposition of coating [63].

However, the results of tensile strength tests showed that the strength of the composite materials was invariant to the surface modification of the carbon fibers. The results of experiment are shown in Table 3.

It was shown that the tensile strength of the composites with different fiber content and reinforced with different modified fibers were approximately the same, reaching about 700 MPa. To understand the reasons for the observed phenomenon, mechanical tests of carbon fibers were conducted. The results of the carbon fiber tensile strength tests can be viewed in Table 4.

The mechanical tests showed that the tensile strength of the fibers reduced from 1063 MPa to 900 MPa after thermal oxidation. The oxidation of the amorphous carbon of the CFs decreased the mechanical properties of the fibers and, consequently, the properties of the composites reinforced with them. In this case, the reduction in the tensile strength of the carbon fibers outweighed the improvement in the adhesion between the matrix and the reinforcement, which resulted in a drop in the properties of the composite material.

**Figure 10:**
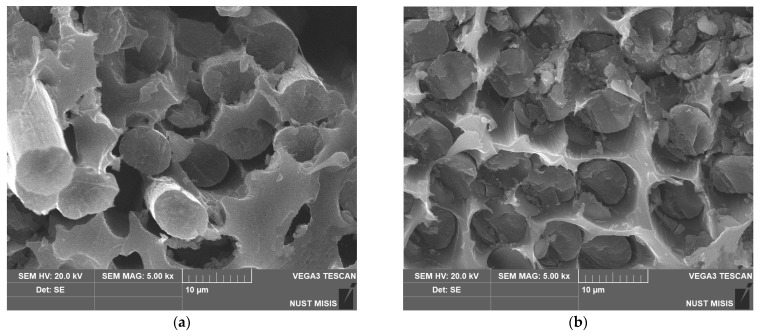

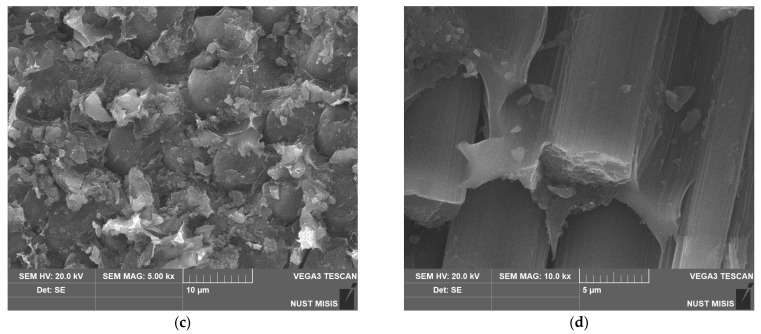
SEM photos of 50/50 composite materials reinforced with (**a**) initial CF, (**b**) TO 300 fibers, and (**c**,**d**) TO 500 fibers.

To investigate the internal structure of the composite materials, scanning electron microscopy was conducted. It was noted that increased adhesion between the matrix and the fibers changed the structure of composite. The results of the SEM are shown in Figure 10.

The SEM images show that the composites reinforced with the initial CFs had an empty space between the polymer matrix and the reinforcement. This phenomenon, which was a result of bad interfacial interactions between the PES and the initial fibers, explains the insufficient mechanical properties of the obtained composites. Empty spaces between the phases of the material serve as stress concentrators, which results in the relatively fast destruction of the material. After surface modification at 300 °C, empty spaces are less notable and reinforcing the composite with TO500 fibers allowed us to fully get rid of the aforementioned stress concentrators. Thus, the SEM study proved the earlier expressed explanations of the mechanisms and the behavior of the composite material.

**Figure 11:**
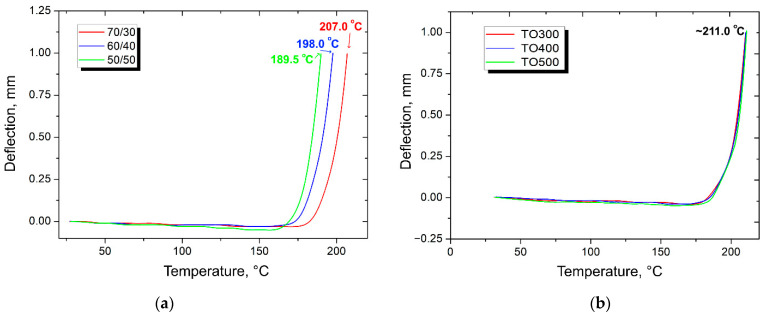
HDT curves of composites reinforced with (**a**) initial CF and (**b**) CF after surface modification (fiber content of 50 wt. %).

Thermophysical properties are another important group of properties for any constructive material. HDT analysis was conducted to understand the thermal limits of use of the composite materials. The results of the study are represented at Figure 11.

After the study, the following results were obtained. For the composites reinforced with the initial CFs, heat deflection temperature raised steadily from the composite with the fiber content of 50 wt. %, at ~190 °C, to the composite with the CF content of 70 wt. %, at 207 °C. Such a tendency can be explained by taking into consideration the thermal stability of carbon fibers in a given thermal span. Since the composites with 70 wt. % contained more CF, the reinforcement allowed the composites to be more stable and rigid at higher temperatures.

Moreover, the surface modification of the carbon fibers resulted in an increase in thermal stability. Thus, the heat deflection temperature of the composites reinforced with modified fibers reached about 211 °C. Notably, there was no difference in HDT with the thermal oxidation temperature of the reinforced material. Such regularity was accounted for by good adhesion between the matrix and the CF. Polymer chains ‘sticking’ to the surface of the fibers decreased their mobility. In addition, as it was shown with the SEM analysis, the internal empty volume of the composite decreased when the material was reinforced with modified fibers. These two factors resulted in the higher thermal stability of the composite materials.

**Figure 12:**
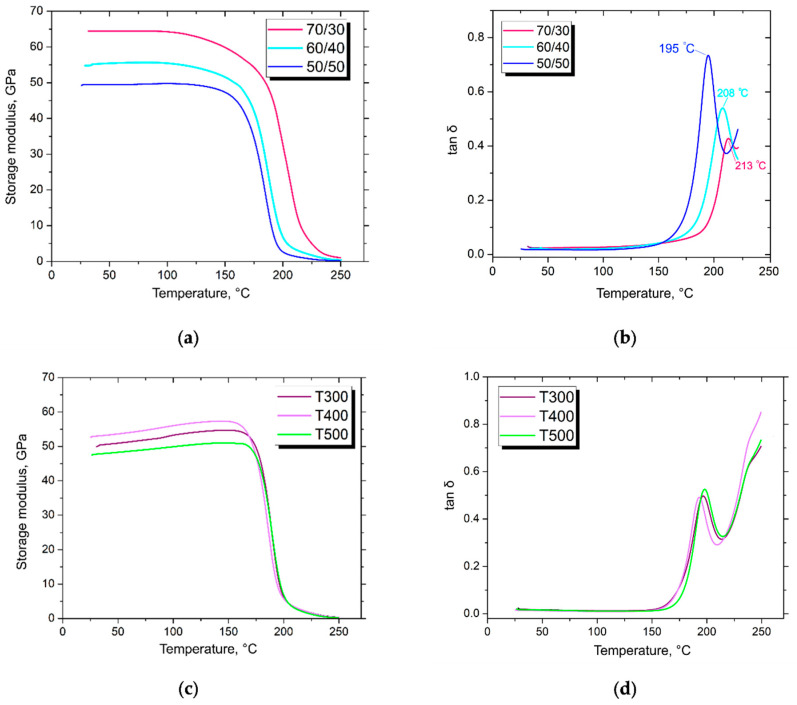
Typical DMA curves of composites reinforced with (**a**,**b**) initial CF, and (**c**,**d**) modified CF (composites reinforced with modified fibers containing 50 wt. % of CF).

DMA was conducted for a more detailed investigation of the thermal properties of the composite materials. Figure 12 represents the results of the analysis.

It has been shown that, generally, all determined thermophysical patterns of obtained composites were the same as during the HDT tests. Thus, the tan δ (loss modulus to storage modulus ratio) was at the maximum point at the temperatures of 195 °C, 208 °C and 213 °C for the composites containing 50, 60, and 70 wt. % initial CF, respectively. Moreover, it has been shown that tan δ was in inverse ratio with fiber content: with the increase in fiber content the maximum value of tan δ lowered. Such a phenomenon can be explained by the lesser thermal stability of polymer matrices with carbon fibers, which results in the loss of the elastic properties of the composites.

The surface modification of carbon fiber influenced the thermal stability of the composites, as well as their viscosity. It was shown that the maximum temperature of thermal stability increased when the peak value of tanδ was significantly lowered, which illustrates the elastic behavior of the composite material during the softening process. Thus, the tan δ of the composites reinforced with initial CF peaked at 0.75, while the maximum value of tan δ for the composites reinforced with modified fibers reached ~0.5. Some studies conducted on polymer-based composite materials have shown that the maximum value of tan δ is influenced by the interfacial interaction between matrix and reinforcement [67]. Therefore, the decrease in tan δ showed better interfacial interaction between the modified fibers and PES. Better adhesion between the phases of the composites limits the thermal mobility of the polymer chains, increasing the viscosity of the polymer matrix and consequently lowering loss modulus.

**Figure 13:**
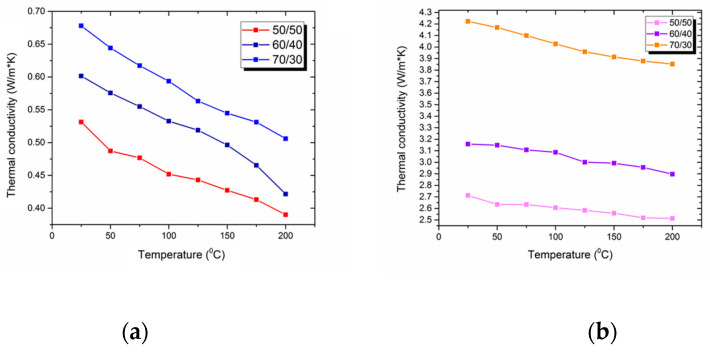
Results of the study of the thermal conductivity of the composites (with initial CF) (**a**) perpendicular to the reinforcing layers of CF and (**b**) along them.

Thermal conductivity is one of the most important thermophysical properties of carbon fiber-reinforced composites. It determines in which conditions composites can be used. Thermal conductivity was investigated with the laser flash technique (LFA), which is considered as an effective method of studying thermal conductivity. Since the obtained composite materials have an anisotropic structure, thermal conductivity was studied in two directions: along the layers of the carbon fibers and perpendicular to them. The results of the experiment are shown in Figure 13.

**Figure 14:**
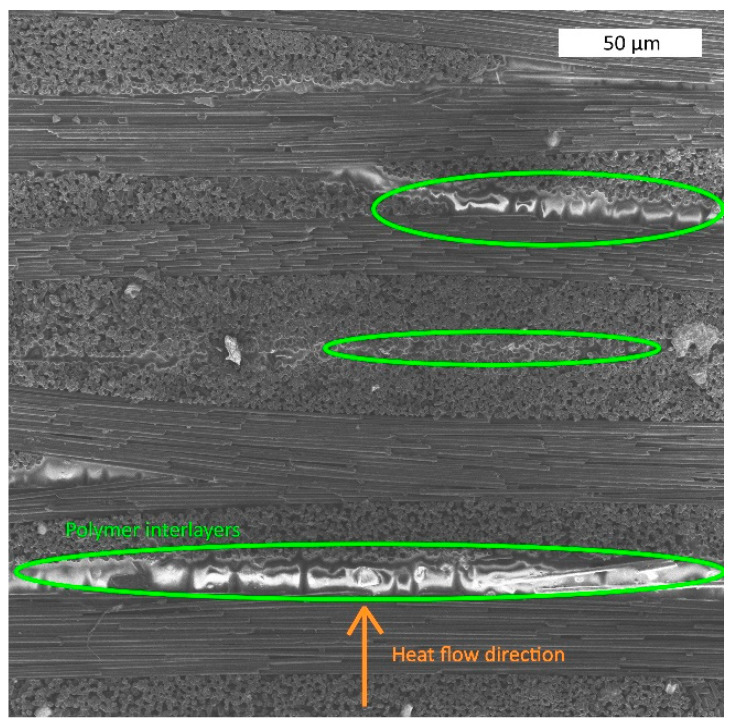
Scheme of heat flow distribution in the investigated composites for the 70/30 composite example.

It was shown that thermal conductivity is strongly influenced by the fiber content and direction of the carbon fibers. Thus, the thermal conductivity (studied perpendicular to the layers of CF at room temperature) of the samples with fiber contents of 70 wt. %, 60 wt. %, and 50 wt. % reached the values of 0.68, 0.6, and 0.53 W/m·K, respectively. The analysis of the results along the layers of reinforced material showed a much higher thermal conductivity of composites. Hence, the coefficients of the thermal conductivity of the composites studied in the layer direction were 4.22, 3.16, and 2.71 W/m·K for the samples with fiber contents of 70, 60, and 50 wt. %, respectively. It is well-known that carbon fibers intensively conduct heat [68], so increasing the amount of carbon fibers explains the corresponding rise in thermal conductivity. Moreover, when the investigated value was studied in the direction perpendicular to the layers of reinforced material, heat flowed through the interlayers of the polymer matrix that did not have properly impregnated carbon fibers (Figure 14). Such interlayers had significantly lower coefficients of thermal conductivity, explaining the observed phenomena. When the direction of heat flow distribution coincides with the direction of the layout, no obstacles in the way of the heat flow are present, which results in a dramatically better release of the potential thermal conductivity of the carbon fibers in the obtained composites. Therefore, considering that the main thermal conductors are carbon fibers, interphase interactions between fibers and polymer matrices may not notably influence the thermal conductivity of the composites. Consequently, such influence was not studied in this research. Considering the thermal conductivity of the carbon fibers as 10 W/(m·K), the release of the initial properties of the reinforced material was about 55–60% for the obtained composites.

Another important feature of any constructive composite material is the coefficient of thermal expansion. The low coefficient of thermal expansion allows the material to stay stable throughout heating. The coefficient of thermal expansion was indirectly investigated by means of TA Instruments Q800. As in the study of thermal conductivity, the thermal expansion of the composite material was studied in two directions. The results of the experiment can be shown in Table 5.

**Figure 15:**
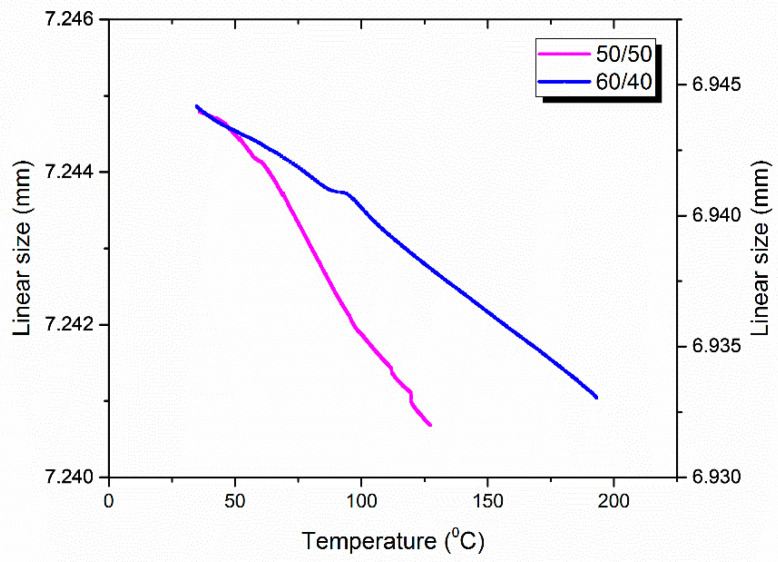
Typical CTE curves investigated in the direction parallel to the layers of reinforcement.

It is noteworthy that CTE expansion is strongly influenced by fiber content. The difference in the thermal expansion coefficient of carbon fibers and the polymer matrix allows us to manage its value by changing the fiber content of the composite material. Thus, the coefficient of the linear expansion of the composites reinforced with 70 wt. % fibers reached the value of 2.7 × 10^−5^ mm/(m × °C). Moreover, in the direction parallel to the axis of reinforcement, CTE became negative (Figure 15). Such phenomena can be explained by the “negative CTE” of carbon fibers when they shrink along their axis with the rise in temperature [69]. Thus, the coefficient of thermal expansion for the 50/50 composite was about −0.69 × 10^−5^ mm/(m × °C).

According to the results of the study of CTE, it is potentially possible to develop and obtain composite material of a particular fiber content in which CTE is close to zero in the investigated range of temperatures.

## 4. Discussion

During the study, the mechanical and thermophysical properties of carbon fiber-reinforced composite materials based on polyethersulfone were investigated. Moreover, the influence of the surface modification of carbon fibers via thermal oxidation on the mechanical and thermophysical properties of the composites was investigated.

X-ray spectroscopy of the surfaces of the carbon fibers showed that the surface modification of the carbon fibers resulted in changes in the chemical composition of the surfaces of the fibers. An increased quantity of C atoms on the surface of the fibers was considered as an indicator of the destruction of the coupling agent on the surface. Moreover, an extended quantity of types of chemical bonds could be referred to as the decomposition of the coupling agent as well.

Atomic force microscopy allowed us to investigate changes in the morphology of the surface. It was shown that several changes in the structure of the surface occurred. Thus, after thermal oxidation at 400 °C, some irregularities on the surface of the fibers appeared, and were interpreted as the coagulation and decomposition of the coupling agent. Therefore, considering the results of the X-ray spectroscopy and AFM, it is concluded that the surface modification of carbon fibers via thermal oxidation could change the chemical composition and morphology on the surface of the CFs.

Because of the changes occurring on the surfaces of the carbon fibers during surface modification, the thermal oxidation of CF can dramatically change the properties of the obtained composite materials. Thus, the thermal oxidation of CF can dramatically change the properties of the obtained composite materials. It has been shown that composites reinforced with modified (TO500) fibers have a flexural strength of about 960 MPa and an E-modulus of 60 GPa. For composites reinforced with initial fibers (fiber content 50 wt. %), the same values were ~600 MPa and ~50 GPa, respectively. The interlaminar shear strength test was interpreted as the measure of adhesion between the polymer and the reinforcement. Surface modification increased the shear strength of the composites by 50%, reaching the values of 40 MPa. However, thermal oxidation lowered the mechanical properties of the carbon fibers, which consequently resulted in a low tensile strength of the composite materials, at about 700 MPa.

Regarding the thermophysical properties, the heat deflection temperature of the composites reinforced with modified fibers was ~211 °C. Increased thermal stability was explained by sufficient interfacial interaction between the polymer chains and the surfaces of the CFs, which led to the lower mobility of the chains and consequently to better thermal stability. Moreover, DMA showed that the behavior of heat softening changed depending on the interfacial interaction in the composites. The composites reinforced with modified fibers showed lower values of tan δ, which can be interpreted as further evidence of improved adhesion between PES and CF.

The thermal conductivity of the composites with different fiber contents of reinforced initial fibers was studied. As the obtained composite materials had an anisotropic structure, thermal conductivity was strongly influenced by the investigated direction. Thus, thermal conductivity coefficients were studied in the directions parallel to the layers of reinforcement and perpendicular to them. It was shown that, in both directions, the best thermal conductors were the 70/30 samples. The observed phenomena is a consequence of the higher thermal conductivity of carbon fibers compared with the polymer matrix, leading to the rise in the thermal conductivity of the composite with the increasing quantity of carbon fibers. Moreover, the obtained composites conducted heat flow more efficiently when co-axial to the carbon fiber direction. The suggested explanation for such behavior consists of the hypothesis that heat flow is distributed continuously by CF without facing the less conductive areas of the polymer.

The coefficient of thermal expansion also depended on the fiber content. Since carbon fibers have so-called negative CTE parallel to their axis direction, the composite materials shrank when co-axial to the reinforcement direction. This factor determines the possibility of the development of materials based on the described composites with geometry, invariant to heat.

## 5. Conclusions

In this study, the mechanical and thermophysical properties of carbon fiber-reinforced composite materials based on polyethersulfone were studied. The influence of the surface modification of CF on the mechanical properties, structure, and thermal stability of the obtained composites was investigated. It was shown that surface modification greatly increased the adhesion between PES and CF, and as a consequence increased the mechanical properties of the composites and their heat deflection temperatures, and reduced the peak values of tan δ, indicating a lower mobility of the polymer chains bonded with the modified surfaces of CFs.

Thermal conductivity and the CTE of composites reinforced with initial fibers in different directions were studied. The dependence of these properties on fiber content and direction was described.

Since there are few studies that investigate PES-based composites reinforced with carbon fabrics, this study represents relevant and important findings that are of special interest.

## Figures and Tables

**Table 1:** Results of flexural strength tests.

Fiber Content, wt. %	Shear Strength, MPa	E-Modulus, GPa
50	602 ± 23	50 ± 4
60	540 ± 30	58 ± 3
70	482 ± 28	66 ± 5

**Table 2:** Results of interlaminar shear strength tests.

Fiber Content, wt. %	Shear Strength, MPa
50	20 ± 3
60	16 ± 3
70	15 ± 3

**Table 3:** Results of tensile strength test (composites reinforced with modified fibers containing 50 wt. % of CF).

Composite Material	E-Modulus, GPa	Tensile Strength, MPa
50/50	45.1 ± 2.0	688.33 ± 24.6
60/40	47.3 ± 0.5	658.5 ± 20.5
70/30	57.8 ± 1.1	705 ± 18.4
TO 300	49.2 ± 2.2	705.7 ± 26.4
TO 400	52.4 ± 3.0	708.3 ± 21.6
TO 500	47.6 ± 23	707.0 ± 25.6

**Table 4:** Results of tensile strength tests of carbon fibers.

Carbon Fibers	Tensile Strength, MPa	Relative Elongation, %
Initial	1063.2 ± 40.1	3.2
TO 300 °C	802.9 ± 47.0	2.7
TO 400 °C	898.8 ± 47.5	3.1
TO 500 °C	898.5 ± 55.1	3.4

**Table 5:** Thermal expansion of composite materials reinforced with initial CF, measured perpendicular to the layers of carbon fibers.

Fiber Content, wt. %	Coefficient of Linear Expansion, mm/(m × °C)
Pure PES	6.3 × 10^−5^
50	4.9 × 10^−5^
60	4.3 × 10^−5^
70	2.7 × 10^−5^

## Data Availability

Not applicable.
